# Does one of the two most commonly used scoring systems have a decisive advantage over the other in diagnosing acute appendicitis in pregnant women?

**DOI:** 10.1097/MD.0000000000033596

**Published:** 2023-04-28

**Authors:** Osman Bardakçi, İbrahim Burak Bahçecioğlu, Faik Tatli, Abdullah Özgönül, Muhammet Emin Güldür, Ali Uzunköy

**Affiliations:** a Department of Surgical Oncology, Ankara Gülhane Research and Training Hospital, Ankara, Turkey; b Department of General Surgery, Harran University Faculty of Medicine, Sanliurfa, Turkey; c Department of Pathology, Harran University Faculty of Medicine, Sanliurfa, Turkey.

**Keywords:** acute appendicitis, Alvarado Score, Appendicitis Inflammatory Response Score, pregnancy

## Abstract

This study aimed to compare the accuracy and reliability of Alvarado Score (AS) and Appendicitis Inflammatory Response Score (AIRS) in pregnant women undergoing surgery for acute appendicitis (AA). The files of 53 pregnant women with a diagnosis of AA who underwent surgery in our clinic between February 2014 and December 2018 were examined retrospectively. The patients were divided into 3 groups as follows: first trimester between 0 and 14 weeks, second trimester between 15 and 28 weeks, and third trimester between 29 and 42 weeks. The AS and AIRS values were calculated according to preoperative physical examination and laboratory results. The mean age of the patients was 28.58 (18–44) years. According to the pathology results, appendicitis was detected in 16 of 23 patients in the first trimester, in 22 of 25 patients in the second trimester, and in 2 of 5 patients in the third trimester. The AIRS was ≥ 9 in 9 patients and the AS was ≥ 7 in 19 of the 23 patients in the 1st trimester, while the AIRS was ≥ 9 in 11 patients and the AS was ≥ 7 in 19 of the 25 patients in the 2nd trimester. However, in the 3rd trimester, the AIRS was ≥ 9 in 2 patients and AS was ≥ 7 in 4 of the 5 patients. In conclusion, when the data obtained from the present study were evaluated, it was determined that both AS and AIRS are effective methods for diagnosing AA in pregnant women.

## 1. Introduction

Acute appendicitis (AA) is the most common cause of urgent non-obstructive surgery in pregnant women, and its incidence ranges from 1.8 to 41 per 10,000 pregnancies.^[1]^ AA may occur at any stage during pregnancy^[2]^; however, the diagnosis of this disease is difficult because of nonspecific abdominal symptoms during pregnancy, physiological leukocytosis, and changes in the anatomical localization of the appendix. Accordingly, the incidence of maternal and fetal complications has increased.^[3]^ The risk of preterm delivery and fetal loss rates in complicated cases were 11% and 6%, respectively, in patients without complications 6% and 2%, respectively.^[4]^ Therefore, it is important to diagnose and treat AA during pregnancy before the occurrence of any complications.^[5]^

Different scoring systems that use multiple parameters have been used to predict the diagnosis of AA. For example, the Alvarado Score (AS) and Appendicitis Inflammatory Response Score (AIRS) are the 2 most accepted and widely used scoring systems.

AS is the most well-known scoring system for the diagnosis of AA and was first described in 1986. AS was calculated according to the patient’s anamnesis, examination findings, and laboratory results. The AS system consists of 8 parameters, with a maximum score of 10. Surgical consultation is recommended for the decision to operate because of AA when the AS is ≤ 7 points; patients who receive a score between 7 and 10 on the AS are recommended for surgery, while those with scores of 5 to 6 are recommended for evaluation using additional methods (Table 1).^[6]^

**Table 1:** Alvarado scoring system.

Parameter	Score
**Abdominal pain**	1
**Anorexia**	1
**Nausea or vomiting**	1
**Muscular guarding in the right lower quadrant**	2
**Temperature > 37.3°C**	1
**Rebound tenderness referred to right lower quadrant**	1
**WBC > 10,000**	2
**Left shift of polymorphonuclear WBC forms > 75%**	1

AIRS is another well-known scoring system that can be used for AA and was first described in 2008. The AIRS includes 7 parameters, with the highest score of 12. The AIRS includes parameters similar to those of AS, but it also has additional parameters, such as C-reactive protein (CRP) (Table 2). Patients with a total score of 9 or above, as calculated according to the parameters of the AIRS system, should be operated on because of the high probability of appendicitis.^[7]^

**Table 2:** Appendicitis inflamatuvar responce scoring system.

Parameter	Score
Vomiting	1
Pain in RIF	1
Rebound tenderness	
Light	1
Medium	2
Strong	3
Temperature ≥ 38.5°C	1
Polymorphonuclear leukocytes	
70–84%	1
≥85%	2
Leukocytes	
>10.0–14.9 × 10^9^/L	1
≥15.0 × 10^9^/L	2
CRP	
10–49 mg/L	1
≥50 mg/L	2

CRP = C-reactive protein, RIF = right inferior fossa.

This study aimed to compare the accuracy and reliability of AS and AIRS in pregnant women undergoing surgery for acute appendicitis.

## 2. Materials and methods

This study was approved by the local ethics committee of the Harran University Medical Faculty in Turkey (approval number: 07.06.2018-14). The files of 53 pregnant women with a diagnosis of AA who underwent surgery in our clinic between February 2014 and December 2018 were examined retrospectively. Patient age, laboratory values, physical examination findings, and pathological results were recorded. Pregnancy in weeks was calculated according to the date of menstruation. The patients were divided into 3 groups as follows: first trimester between 0 and 14 weeks, second trimester between 15 and 28 weeks, and third trimester between 29 and 42 weeks. The AS and AIRS values were calculated according to preoperative physical examination and laboratory results. AS was divided into 2 groups, <7 and ≥7, and AIRS was divided into groups of <9 and ≥9. We used a checklist to assess the migration of pain, anorexia, nausea, tenderness, and rebound pain. If the file did not include these data, we excluded the patients. Patients in both groups treated with open surgery underwent appendectomy through a McBurney incision.

### 2.1. Statistical analysis

Statistical Package for the Social Sciences, version 18 (SPSS Inc., Chicago, IL) was used for statistical analyses. Numerical data are presented as mean ± standard deviation. The one-sample Kolmogorov–Smirnov test was used to evaluate the distribution of the numerical data. The independent samples *t* test was used in cases in which the distribution was normal, whereas the Mann–Whitney *U* test was used in cases in which the distribution was not normal. The chi-square test was used for comparisons between groups. Statistical significance was set at *P* < .05.

## 3. Results

A total of 53 pregnant women who underwent surgery for AA were included in this study. The mean age of the patients was 28.58 (18–44) years. According to the pathology results, appendicitis was detected in 16 of 23 patients in the first trimester, in 22 of 25 patients in the second trimester, and in 2 of 5 patients in the third trimester. The AIRS was ≥ 9 in 9 patients and the AS was ≥ 7 in 19 of the 23 patients in the 1st trimester, while the AIRS was ≥ 9 in 11 patients and the AS was ≥ 7 in 19 of the 25 patients in the 2nd trimester. However, in the 3rd trimester, the AIRS was ≥ 9 in 2 and the AS was ≥ 7 in 4 of the 5 patients (Table 3).

**Table 3:** AS, AİRS, Distribution of pathology results according to trimesters.

		1. Trimester	2. Trimester	3. Trimester
AS	≥7	21	20	4
	<7	2	5	1
AİRS	≥9	14	19	2
	<9	9	6	3
Pathology	Appendicitis	16	22	2
	Non-appendicitis	7	3	3

AIRS = Appendicitis Inflammatory Response Score, AS = Alvarado Score.

When the scores were compared without distribution according to the trimesters, but according to the pathology results of the patients who underwent appendectomies, appendicitis was detected in 3 (37.5%) patients with an AS value of < 7, while the appendix was normal in 5 (62.5%) patients. While 37 (82.2%) patients with an AS value ≥ 7 had appendicitis, the appendix was found to be normal in 8 (17.8%) patients. Of the 18 patients with AIRS values < 9, 6 (33.3%) had pathological appendicitis, while 12 (66.7%) did not. Of the 35 patients with AIRS values ≥ 9, 34 (97.1%) had pathological appendicitis, while 1 (2.9%) did not (Table 4).

**Table 4:** Comparison of AS, AİRS, and CRP values according to pathology results.

		Pathology Appendicitis	Pathology Non-appendicitis	*P*
AS	<7	3 37.5%	5 62.5%	
	≥7	37 82.2%	8 17.8%	.007
AİRS	<9	6 33.3%	12 66.7%	
	≥9	34 97.1%	1 2.9%	.001
CRP (mg/dL)		2.27 ± 3.21	7.80 ± 10.19	.009

AIRS = Appendicitis Inflammatory Response Score, AS = Alvarado Score, CRP = C-reactive protein.

When the CRP values of the pregnant women undergoing appendectomies were compared according to the pathology results, the mean CRP value of the patients with appendicitis was 7.80 ± 10.19 mg/L, while the mean CRP value of the patients without appendicitis was 2.27 ± 3.21 mg/L. There was a statistically significant difference between the CRP levels (*P* < .009) (Table 4).

## 4. Discussion

Our research aims to define the efficiency of 2 scoring systems that are most commonly used in the world for acute appendicitis diagnosis in pregnant societies by comparing pathology results. Currently, no scoring system specifically evaluates appendicitis during pregnancy. Therefore, we evaluated the efficiencies of the AS and AİRS systems.

AA is diagnosed using clinical and physical examinations, and ancillary diagnostic methods. However, the diagnosis of AA is more complex because of the physiological and anatomical changes that occur during pregnancy. As a result, maternal and fetal mortality and morbidity rates have increased. Delays in the diagnosis and treatment of AA can cause spontaneous abortion, preterm birth, low birth weight, and mortality during the first 7 days.^[8]^ The most common surgical cause of fetal loss during pregnancy is a patient showing complications due to appendicular perforation due to a delay in the diagnosis.^[9]^

CRP is an acute-phase reactant synthesized in the liver and is a useful parameter for diagnosing and monitoring acute and chronic inflammatory conditions. CRP levels may increase in healthy pregnant women.^[10]^ Moreover, high CRP levels are not specific for AA in pregnant women. However, when AA is suspected, it may be useful to support the diagnosis.^[11]^ In a study conducted by Jung et al on pregnant patients presenting with acute abdominal pain, it was determined that surgical treatment was needed in patients with CRP values higher than 1.82 mg/dL.^[12]^ In the present study, when the CRP values were compared according to the pathology results, there was a statistically significant difference (*P* = .009).

Laboratory tests used in the diagnosis of AA are not reliable in pregnant women because of the occurrence of physiological changes. Jung et al suggested that surgical treatment may be necessary for patients with white blood cell values greater than 11,000/mm^3^ and neutrophil percentages of over 79.9% among pregnant women presenting with acute abdominal pain.^[12]^ In AS, the white cell value was 2 points when it was above 10,000/mm^3^, and a shift to the left was 1 point when it was above 75%. In the AIRS, the white cell value is 1 point from 10,000–14,900/mm^3^ and 2 points when it is above 15,000 mm^3^, whereas a shift to the left is 1 point from 70% to 84% and 2 points when it is ≥85%. Therefore, these scoring methods may be useful for diagnosing AA in pregnant women. The aim of a clinical scoring system in patients with suspected AA is not to diagnose primary appendicitis but to determine the likelihood of AA, select appropriate patients for urgent surgery, and assist in patient selection for further monitoring and observation with additional imaging methods.^[13]^

AS has been shown to be highly valid when applied to populations in the United States and Europe.^[14]^ In the study conducted by Tatli et al,^[15]^ it was reported that the AS is an easy-to-use and noninvasive ancillary diagnostic tool with high diagnostic accuracy in patients with suspected AA. Parallel to this, in the study by Augustin et al patients with a score > 7 were associated with 82.9% sensitivity and 86.4% specivity.^[16]^ Although the Alvarado system may be used in pregnant patients, its use has been extensively validated in non-pregnant patients.^[17]^ In the present study, pathological appendicitis was detected in 82.2% of patients with AS values ≥ 7, while this rate was 37.5% in patients with AS values < 7.

AİRS; while it is based on values similar to those of AS, it also contains CRP as an additional new variable.^[7]^ Although the RİPASA system performed the best in Mantoglu et al’s research that compared 9 different appendicitis scoring systems in pregnant society, its sensitivity and specificity were under 80%. In the same study, the sensitivity and specificity found as 80% and 71.4%, AS’s sensitivity and 58.5% and 78.6%.^[18]^ In the present study, appendicitis was detected pathologically in 97.1% of pregnant patients with AIRS values ≥ 9, whereas this rate was 33.3% in pregnant patients with AIRS values < 9.

Our study had some limitations. First, due to our research’s retrospective features, we have limited examination data. Second, the number of patients (especially in 3th trimester) was low. The strength of our study is that it was conducted in a third-stage hospital and at a single center.

## 5. Conclusion

In conclusion, when the data obtained from the present study were evaluated, it was determined that both AS and AIRS are effective methods for diagnosing AA in pregnant women. However, the fact that the AIRS containing the CRP value has higher accuracy suggests an advantage for this system.

## Acknowledgments

We are grateful to all patients and doctors contributed to this paper.

## Author contributions

**Conceptualization:** Osman Bardakçi, Faik Tatli.

**Data curation:** Osman Bardakçi, İbrahim Burak Bahçecioğlu, Muhammet Emin Güldür.

**Formal analysis:** Osman Bardakçi, İbrahim Burak Bahçecioğlu, Muhammet Emin Güldür.

**Investigation:** Ali Uzunköy.

**Methodology:** Faik Tatli, Ali Uzunköy.

**Project administration:** Osman Bardakçi, Ali Uzunköy.

**Resources:** Osman Bardakçi.

**Software:** Muhammet Emin Güldür, Ali Uzunköy.

**Validation:** Osman Bardakçi.

**Visualization:** Osman Bardakçi.

**Writing – original draft:** Osman Bardakçi, Ali Uzunköy.

**Writing – review & editing:** Faik Tatli, Abdullah Özgönül, Ali Uzunköy.
